# Neighborhood green spaces, facilities and population density as predictors of activity participation among 8-year-olds: a cross-sectional GIS study based on the Norwegian mother and child cohort study

**DOI:** 10.1186/s12889-019-7795-9

**Published:** 2019-10-30

**Authors:** Emma Charlott Andersson Nordbø, Ruth Kjærsti Raanaas, Helena Nordh, Geir Aamodt

**Affiliations:** 1 Department of Public Health Science, Faculty of Landscape and Society, Norwegian University of Life Sciences, PO Box 5003, NO-1432 Ås, Norway; 2 The Centre for Evidence-Based Public Health, A Joanna Briggs Institutes Affiliated Group, Ås, Norway

**Keywords:** Built environment, Geographic information systems, Children, Physical activity, Organized activity, Social activity, Well-being, The Norwegian mother and child cohort study

## Abstract

**Background:**

A rapidly growing body of research suggests that qualities of the built environment can promote active living among children and youth. Nevertheless, shortcomings in the current evidence for understanding which built environment characteristics provide opportunities for taking part in activities in childhood remain. This study aimed to examine whether population density, green spaces, and facilities/amenities are associated with participation in leisure-time physical activity (PA), organized activities, and social activities with friends and peers in Norwegian 8-year-olds.

**Methods:**

Data from a sample of 23,043 children from the Norwegian Mother and Child Cohort Study (MoBa) were linked with geospatial data about the built environment. The questionnaire data reported by mothers provided information on the children’s leisure activities. We computed exposure to neighborhood population density and access to green spaces and facilities/amenities within 800- and 5000-m radii of the participants’ home addresses using geographic information systems. Associations were estimated using logistic regression models.

**Results:**

We found beneficial associations between having a park within 800-m and more leisure-time PA during the summer. Furthermore, children living in neighborhoods with higher proportions of green space participated in more PA during the winter. More densely populated areas and access to facilities were associated with participation in organized and social activities. Specifically, we observed that more playgrounds/sport fields in the neighborhood were the strongest and most consistent correlate of activity participation in Norwegian 8-year-olds by being related to more socialization with friends and peers.

**Conclusion:**

This population-based study underscores the importance of access to a variety of venues and opportunities for different activities in the immediate neighborhood surroundings and in the greater community to support participation in physical activity and organized and social activities in childhood.

## Background

Participating in leisure activities is essential for children’s health and well-being [1, 2]. Involvement in different organized activities, such as team and individual sports, music activities, and social clubs, has been associated with increased academic achievement, positive social relationships, higher self-rated health and life satisfaction, and better mental health [2–4]. The health benefits of physical activity in childhood are also widely known and supported [5], and a substantial amount of evidence has highlighted the importance of social activities with friends for physical, psychological, and social well-being [6, 7]. A great amount of time in children’s everyday life is devoted to leisure activities [8], and leisure is therefore an important context for health promotion and well-being enhancement in childhood.

All kinds of activities take place in different settings [9]. According to socio-ecological models of health and active living, neighborhoods are key settings for activities that children can enjoy, in particular during leisure-time [10]. To ensure children have opportunities to engage in leisure activities that can promote their well-being, knowledge about built environment characteristics of neighborhoods, and whether they facilitate activity participation, is important for different stakeholders [11]. It has been proposed that the neighborhood environment may exert influence on children’s leisure activities by providing resources essential for participation [12, 13].

A growing body of research has identified characteristics of the built environment that seem to promote active living among children and adolescents [14–16]. Studies reported that neighborhoods with high walkability, low traffic exposure and high safety, pedestrian infrastructure for walking and cycling, and access to facilities support active travel [15, 17, 18]. Furthermore, built environments characterized by mixed land-use, versatile facilities (e.g., a local community center that are adapted to host several activities), high street connectivity, and direct pedestrian access may promote physical activity; however, these findings are more inconsistent and inconclusive [16, 19]. In addition, there is evidence that access to green space and safety from traffic and crime are related to children’s outdoor play [20]. Some studies have linked shorter distances to green spaces and recreation facilities (such as sports fields, swimming pools, and parks) to increased participation in sport activities [21, 22]. Several studies have also shown that densely populated areas are associated with higher levels of physical activity [23, 24] and outdoor activity [25] compared to less populated areas. However, other studies examining these associations did not report the same results [26–28].

Nevertheless, we see several shortcomings in the evidence for understanding the ways in which the built environment provides opportunities for participation in various leisure activities in childhood. First, the majority of the studies mentioned above focused on physical activity or active travel. Besides physical activity, children may benefit from involvement in many other activities, such as organized and social activites with friends and peers, which are common activities during leisure-time among Norwegian children [29, 30]. These potentially well-being enhancing activities are important to consider as the neighborhood built environment may exert influence on all of them. Structural characteristics, such as population and residential density, are regarded as potential predictors of activity participation because neighborhood areas with higher density generally have more facilities and may therefore create more opportunities for taking part in a variety of leisure activities and for socializing [14, 31, 32]. Moreover, it has been proposed that neighborhood green space is likely to be an attractive setting in which to conduct physical activity and meet other people [33]. Limited research has addressed whether these built environment characteristics act as potential facilitators for participation in a more extensive variety of leisure activities. Increased knowledge on this matter can represent a valuable contribution to inform how we can create health-promoting neighborhoods for children. Second, previous studies focused on children or adolescents older than 10 years, whereas less attention has been paid to the influence of the built environment on 5- to 8-years-olds’ activities [19]. From a holistic public health and developmental perspective, 8-year-olds are interesting because relationships with friends and peers are extremely important in this phase of social development, and participation in sports and group activities is highly appreciated. Although children’s degrees of freedom to move around independently have diminished during the last decades [34], children at this age are also increasingly getting their parents’ permission to explore new territory and expand their spatial world [35]. As such, more research examining nearby activity venues that children may use without parental supervision is important, particularly since the independent mobility levels of Norwegian children rank high in international comparisons [34]. Likewise, since young children still largely depend on adult accompaniment to take part in leisure activities, it is also vital to obtain more knowledge about available facilities and activity venues in the greater community. Third, few large-scale studies that include children across diverse geographical areas have been conducted. Use of geographic information systems (GIS) facilitates the examination of objectively measured built environment determinants in large population studies [36, 37]. There has been a call for more studies from a broader range of countries that use GIS-derived measures to examine relations between the built environment and children’s health and well-being [19]. To address these gaps, the aim of this study was to examine whether the built environment characteristics population density, green spaces, and facilities are associated with participation in leisure-time PA, organized and social activities with friends in a large and geographically diverse sample of 8-year-old children in Norway.

## Methods

### Study design and data sources

In this study, we applied a cross-sectional design in which data from the Norwegian Mother and Child Cohort Study (MoBa) were linked to geospatial data about the built environment around the participants’ home addresses. MoBa is a nationwide prospective population-based pregnancy cohort conducted by the Norwegian Institute of Public Health. Detailed descriptions of the cohort have been published elsewhere [38]. Pregnant women were recruited from all over Norway during the years 1999–2008. Of the eligible mothers, 41% consented to participate. The cohort comprises 95,200 mothers and 114,500 children. The present study is based on version IX of the quality-assured data files released for research in November 2015. We used the 8-year follow-up surveys completed by the mothers and obtained available data from those children who turned 8-years old in 2011, 2012, 2013, 2014 and 2015.

### Subjects

**Fig. 1:**
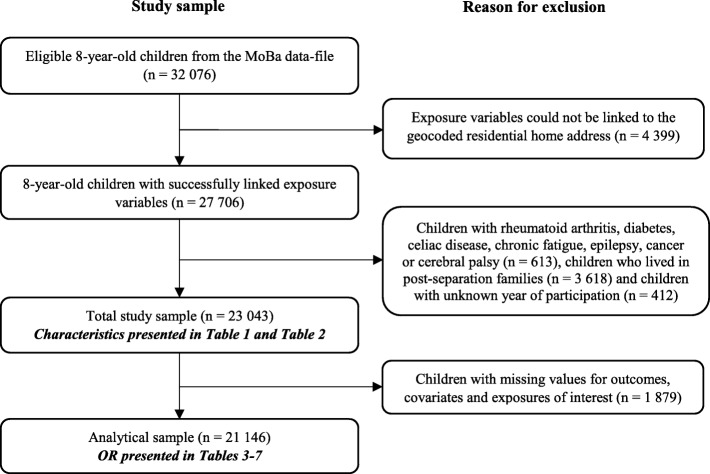
Participant flow diagram

Questionnaire data reported by mothers were available for 32,076 children at the time of access. To be included in this study, the exposure variables had to be successfully linked to each participant’s geocoded residential address. We excluded children with specific diagnoses. Children living in post-separation families were also excluded as the exposures were computed around the mothers’ addresses only. Additionally, we excluded children with unknown year of participation in the follow-up, resulting in a total sample of 23,043 children. All these children turned 8-years old between 2011 and 2015. We removed participants with missing data for key variables, and consequently, 21,146 eight-year-olds were included in the analytical sample. The participant flow diagram is displayed in Fig. 1.

### Outcome variables

The questionnaire provided information about the child’s leisure activities, friends, and general health, as well as demographic information of the mother [39]. Outcome variables and covariates were derived from this material.

To measure *leisure-time PA* we used two questions to elicit how much time the child spent on physical activity outside school hours during the summer and winter. The selectable six options were “< 1,” “1–2,” “3–4,” “5–7,” “8–10,” and “≥ 11” h/week. We recoded the answers into a dichotomous variable “≥5 h/week” opposed to “4 ≤ h/week,” a threshold that concurs with recommendations from the Norwegian health authorities of 60 min/day (7 h/week) of moderate-to-vigorous PA [40]. The remaining hours of physical activity would be expected to occur at school, during either recess or physical education classes.

To measure *participation in organized activities*, one question addressed how many days per week the child participated in any kind of organized leisure activity (e.g., sport, music, or theater). The response categories were “never/seldom,” “once a week,” “2–3,” “4–5,” and “6–7 days/week”. The answers were recoded into a dichotomous variable: “2 days or more/week” instead of “once a week or less” based on that Norwegian children on average participate in 1.7 organized activities [41].

We measured *informal social activity with friends and peers* with a question that elicited how many days per week the child spent time with friends and peers, excluding school hours and organized activities. This outcome variable was dichotomized into the categories “2 days or more/week” and “once a week or less.” This threshold is grounded in surveys showing that nearly 60% of Norwegian children spend time with their friends at least twice a week [30].

### Assessment and linkage of exposure variables

To calculate exposure to population density, facilities/amenities and neighborhood green space, we used GIS (ArcGIS 10.3 and QGIS 2.14). We downloaded geographic data from 2016 and up until January 2017. The built environment exposures were calculated within 800- and 5000-m circular buffers of the geo-referenced residential addresses. The smaller radius represented the neighborhood surroundings, and the larger radius represented the greater community. The 800-m radius was selected based on previous research showing that this spatial unit has been most frequently applied in existing studies and seems to capture the neighborhood areas children use for activity purposes [37, 42]. The 5000-m radius was chosen as it was thought that facilities spread across larger geographical areas were important to examine for the following reasons: (1) the Norwegian context characterized by low centrality in many areas [43], (2) the diminished degrees of freedom that children have to move around independently indicate that parents are accompany their children to leisure activities; and (3) organized activities are not necessarily undertaken in the neighborhood area of 800-m as such activites usually are directed by adults [1]. Statistics Norway linked the exposure data to each child in MoBa.

### Operationalization of the built environment determinants

#### Population density

We used the Statistical Grid Dataset (250-m × 250-m) with population data from 2016 from Statistics Norway to assess the population density. Population density was operationalized as the total number of residents per square kilometers around the residential home address of each child. Due to the high computational burden, we calculated this measure within the 800-m radius only. We divided the variable into four categories: ≤ 200 residents (reference), 201–799 (low), 800–1649 (moderate) and ≥ 1650 (high). The quartiles were derived statistically while taking into account the Statistics Norway’s definition of densely populated areas, which states that such areas are characterized by settlements > 200 inhabitants where the distance between the houses does not exceed 50 m [44].

#### Facility and amenity measures

We used the national building and land-use datasets, provided by the Norwegian Mapping Authority, to capture facilities/amenities within the two zones of the residential home addresses. We calculated the total number of facilities/amenities that could serve as potential venues for the studied activities, including schools, libraries, churches, cinemas, indoor pools, shopping malls, and community centers. We also computed the total number of playgrounds/sports fields. Both variables were divided into quartiles. Additionally, we calculated access to school within the zones, which was dichotomized into the “presence of a school” (yes/no).

#### Neighborhood green space

We used national land-cover and land-use datasets to calculate the total area (square kilometers) of green spaces within the defined radii and applied two different measures. For measuring total green space, we considered forests, marshland, parks, and golf courses. Due to the high computational burden, we calculated this measure within the 800-m radius only. We converted the area of green space into the proportion of the total area within the zone and then split the variable into quartiles. We also calculated a separate measure for parks within 800- and 5000-m of the participants’ home addresses. Parks were defined according to the Norwegian Mapping Authority as built-up and maintained green areas larger than 2000 m^2^ and wider than 30 m, with lawns, plants, water features, seating, etc. We dichotomized this measure into the “presence of a park” (yes/no) within the defined radii.

### Covariates

We selected potential confounders a priori based on previous studies and directed acyclic graphs, depicting the links between the variables (see Additional file 1: Figure S1). The following individual-level covariates were adjusted for in the analyses: child’s sex, mother’s age and level of education, and after-school care. In trying to account for potential urban and rural differences, we treated population density as an area-level confounder in addition to considering it as a predictor for activity participation.

### Statistical analyses

Differences in frequencies between the genders were examined using the standard chi-square statistics. We used logistic regression to model the odds of participating in different activities dependent on the built environment exposures and we fitted crude and adjusted models. In the adjusted models, we considered only predictors that were statistically significantly related to participation in activities (*p* < 0.05) in the crude models. As including multiple environmental variables in statistical models can provoke multicollinearity, we computed Spearman’s rho and the variance inflation factor (VIF) before we fit the regression models. The correlation coefficients revealed that population density, facilities/amenities (the 5000-m radius) and playgrounds/sports fields (800- and 5000-m radii) were highly correlated (rho > 0.7). Similarly, the VIF values of the variables exceeded 2.5, indicating potential multicollinearity [45].

To remedy this problem, we estimated separate adjusted odds ratios (ORs) for each built environment exposure. We performed the adjustment in two steps. First, we simultaneously added and adjusted for all individual-level covariates. Next, we added population density with the individual-level covariates. We adjusted for population density only in the absence of multicollinearity between population density and the particular environmental exposure of interest. Researchers have previously reported differences between boys and girls in environmental supportiveness for physical activity [24, 46], and all analyses were stratified according to sex. Finally, we conducted a sensitivity analysis on a sub sample of children (*n* = 8311) who participated in the 8-year follow-up in 2014 and 2015 to assess the robustness of the results.

We reported the odds ratios and the corresponding 95% confidence interval (CI), as well as *p*-values for the trend resulting from models in which the exposures were treated as continuous variables. All analyses were performed using IBM SPSS Statistics 25, and we considered *p*-values less than 0.05 to be statistically significant.

## Results

### Profile of the participants

Individual-level characteristics are presented in Table 1. Within this sample of 23,043 Norwegian 8-year-olds, there were 11,176 (48.5%) girls. The mothers’ educational attainment was high; 38.8% had more than 4 years of university education. Overall, the children were most active during the summer. Statistically significantly more boys participated in ≥5 h/week of leisure-time PA in the summer and winter compared to girls (*p* < 0.001). The majority of the children participated in organized activities (71.3%) and were together with friends ≥2 days/week (82.8%). The distribution of the built environment exposures is shown in Table 2. We did not observed differences for the exposure variables between the sexes. Among those excluded, there were slightly more girls, the mothers were younger and less educated, and the children participated less in activities compared to the study sample (*p* < 0.05). Additionally, those excluded lived in neighborhoods with higher population density and more facilities in the immediate surroundings of their home (data not shown).

**Table 1:** Individual-level characteristics for all children and by gender from 23,043 MoBa participants

	N (%)			
Characteristics	Total (*n* = 23,043)	Boys (*n* = 11,826)	Girls (*n* = 11,176)	*P*-value^a^
Hours of leisure-time PA (summer)				<0.001
≤4 h/week	8758 (38.0)	3658 (30.9)	5086 (45.5)	
≥5 h/week	14,085 (61.1)	8071 (68.3)	5987 (53.6)	
Missing	200 (0.9)	97 (0.8)	103 (0.9)	
Hours of leisure-time PA (winter)				<0.001
≤4 h/week	11,375 (49.4)	5110 (43.2)	6247 (55.9)	
≥5 h/week	11,457 (49.7)	6597 (55.8)	4837 (43.3)	
Missing	211 (0.9)	119 (1.0)	92 (0.8)	
Participation in organized activities				0.003
Once a week or less	6562 (28.5)	3467 (29.3)	3078 (27.6)	
2 days or more/week	16,430 (71.3)	8333 (70.5)	8073 (72.2)	
Missing	51 (0.2)	26 (0.2)	25 (0.2)	
Informal social activity with friends/peers				0.007
Once a week or less	3627 (15.7)	1934 (16.4)	1684 (15.1)	
2 days or more/week	19,084 (82.8)	9719 (82.2)	9333 (83.5)	
Missing	332 (1.5)	173 (1.4)	159 (1.4)	
After school care				0.516
No	6096 (26.4)	3162 (26.7)	2918 (26.1)	
Yes	16,503 (71.6)	8449 (71.5)	8026 (71.8)	
Missing	444 (2.0)	214 (1.8)	230 (2.1)	
Maternal age (years) at recruitment				0.063
≤29	8967 (38.9)	4679 (39.6)	4288 (38.4)	
≥30	14,035 (60.9)	7147 (60.4)	6888 (61.6)	
Missing	41 (0.2)	0 (0.0)	0 (0.0)	
Maternal level of education				0.731
High school or less	4624 (20.1)	2392 (20.2)	2229 (19.9)	
University ≤4 years	8904 (38.6)	4603 (38.9)	4286 (38.4)	
University > 4 years	8951 (38.8)	4576 (38.7)	4355 (39.0)	
Missing	564 (2.5)	255 (2.2)	306 (2.7)	

Note: PA, physical activity

^a^Results from χ^2^ comparing boys and girls

**Table 2:** Distribution of the built environment exposures for 23,043 children from MoBa

	N (%)			
Built environment exposures	Total (n = 23,043)	Boys (n = 11,826)	Girls (n = 11,176)	*P*-value^a^
Total green and open spaces				0.187
≤13.0% (ref.)	5593 (24.3)	2866 (24.2)	2717 (24.3)	
13.1–29.9%	5664 (24.6)	2846 (24.1)	2811 (25.2)	
30–49.9%	5983 (26.0)	3085 (26.1)	2887 (25.8)	
≥50.0%	5803 (25.2)	3029 (25.6)	2761 (24.7)	
Missing	0 (0.0)	0 (0.0)	0 (0.0)	
Park within 800 m				0.671
No	19,279 (83.7)	9882 (83.6)	9362 (83.8)	
Yes	3764 (16.3)	1944 (16.4)	1814 (16.2)	
Missing	0 (0.0)	0 (0.0)	0 (0.0)	
Park within 5000 m				0.517
No	8493 (36.9)	4384 (37.1)	4097 (36.7)	
Yes	14,550 (63.1)	7442 (62.9)	7079 (63.3)	
Missing	0 (0.0)	0 (0.0)	0 (0.0)	
Number of facilities/amenities 800 m				0.325
0 (ref.)	10,837 (47.0)	5600 (47.4)	5220 (46.7)	
1	4687 (20.3)	2429 (20.5)	2253 (20.2)	
2–3	4542 (19.7)	2311 (19.5)	2219 (19.9)	
≥4	2977 (12.9)	1486 (12.6)	1484 (13.3)	
Missing	0 (0.0)	0 (0.0)	0 (0.0)	
Number of facilities/amenities 5000 m				0.689
≤5 (ref.)	6007 (26.1)	3096 (26.2)	2901 (26.0)	
6–14	5512 (23.9)	2856 (24.2)	2647 (23.7)	
15–29	5257 (22.8)	2665 (22.5)	2582 (23.0)	
≥30	6267 (27.2)	3209 (27.1)	3046 (27.3)	
Missing	0 (0.0)	0 (0.0)	0 (0.0)	
Number of playgrounds/sports fields 800 m				0.355
≤1 (ref.)	3666 (15.9)	1928 (16.3)	1733 (15.5)	
2–5	4002 (17.4)	2058 (17.4)	1935 (17.3)	
6–10	3748 (16.3)	1900 (16.1)	1846 (16.5)	
≥11	11,627 (50.5)	5940 (50.2)	5662 (50.7)	
Missing	0 (0.0)	0 (0.0)	0 (0.0)	
Number of playgrounds/sports fields 5000 m				0.176
≤35 (ref.)	5845 (25.4)	3031 (25.6)	2805 (25.1)	
36–119	5654 (24.5)	2839 (24.0)	2806 (25.1)	
120–419	5690 (24.7)	2965 (25.1)	2716 (24.3)	
≥420	5854 (25.4)	2991 (25.3)	2846 (25.5)	
Missing	0 (0.0)	0 (0.0)	0 (0.0)	
School within 800 m				0.145
No	16,540 (71.8)	8540 (72.2)	7974 (71.3)	
Yes	6503 (28.2)	3286 (27.8)	3202 (28.7)	
Missing	0 (0.0)	0 (0.0)	0 (0.0)	
School within 5000 m				0.363
No	4941 (21.4)	2565 (21.7)	2369 (21.2)	
Yes	18,102 (78.6)	9261 (78.3)	8807 (78.8)	
Missing	0 (0.0)	0 (0.0)	0 (0.0)	
Population density				0.072
≤200 (ref.)	4747 (20.6)	2515 (21.3)	2227 (19.9)	
201–799	6679 (29.0)	3397 (28.7)	3271 (29.3)	
800–1649	5832 (25.3)	3004 (25.4)	2817 (25.2)	
≥1650	5649 (24.5)	2853 (24.1)	2783 (24.9)	
Missing	136 (0.6)	58 (0.5)	78 (0.7)	

^a^Results from χ^2^ comparing boys and girls

### Leisure-time PA during summer and winter

Only a few of the built environment exposures were associated with leisure-time PA in summer and winter in the crude analyses (Table 3). After adjustment for individual-level confounders, children with 2–5 and ≥ 11 playgrounds/sport fields within 800 m had 16 and 20% reduced odds of ≥5 h/week PA during the summer, respectively, compared to children with 6–10 and ≤ 1 playgrounds/sports fields (Table 4). We also found negative associations with leisure-time PA during the winter across all quartiles of playgrounds/sports fields within 800 m. Access to school was related to decreased odds of ≥5 h/week leisure-time PA in the summer, but an additional adjustment for population density removed the association. Only neighborhood green spaces were positively associated with leisure-time PA. In the summer, children with a park within 800 m of their home had 12% higher odds of ≥5 h/week leisure-time PA (*p* < 0.01). Similarly, we found a statistically significant trend of more leisure-time PA during the winter with greater proportions of total neighborhood green space (*p*_*trend*_ = 0.002).

**Table 3:** Crude associations between environmental characteristics and activity participation in all children from MoBa

	All children (*n* = 21,146) Crude OR (95% CI)			
	≥ 5 h/week leisure-time PA (summer)	≥ 5 h/week leisure-time PA (winter)	Organized activities ≥2 days/week	Friends and peers ≥2 days/week
Total green space 800 m				
≤13% (ref.)	1	1	1	1
13.1–29.9%	1.03 (0.96–1.12)	1.14 (1.06–1.23)**	1.02 (0.93–1.11)	0.86 (0.78–0.96)**
30.0–49.9%	1.05 (0.99–1.16)	1.13 (1.05–1.22)**	0.93 (0.86–1.02)	0.93 (0.83–1.03)
≥50.0%	1.08 (0.99–1.16)	1.20 (1.11–1.29)**	0.85 (0.78–0.93)**	0.73 (0.66–0.81)**
P for trend	0.114	0.001	0.019	< 0.001
Park within 800 m				
No (ref.)	1	1	1	1
Yes	1.09 (1.01–1.17)*	1.05 (0.98–1.13)	1.13 (1.04–1.22)**	1.06 (0.95–1.16)
Park within 5000 m				
No (ref.)	1	1	1	1
Yes	0.98 (0.93–1.04)	1.04 (0.98–1.10)	1.19 (1.12–1.26)**	1.42 (1.31–1.53)**
Facilities/amenities 800 m				
0 (ref.)	1	1	1	1
1	0.94 (0.87–1.01)	1.01 (0.94–1.08)	1.20 (1.11–1.30)**	1.30 (1.17–1.43)**
2–3	0.98 (0.91–1.06)	0.99 (0.93–1.07)	1.19 (1.10–1.29)**	1.37 (1.24–1.52)**
≥4	0.94 (0.86–1.02)	0.99 (0.92–1.09)	1.25 (1.13–1.37)**	1.13 (1.01–1.27)*
P for trend	0.349	0.411	< 0.001	0.593
Facilities/amenities 5000 m				
≤5 (ref.)	1	1	1	1
6–14	1.07 (0.99–1.16)	1.05 (0.97–1.13)	1.04 (0.96–1.13)	1.35 (1.22–1.49)**
15–29	1.01 (0.93–1.09)	1.02 (0.95–1.11)	1.16 (1.06–1.26)**	1.49 (1.34–1.65)**
≥30	0.99 (0.91–1.06)	1.07 (0.99–1.15)	1.34 (1.23–1.46)**	1.32 (1.20–1.45)**
P for trend	0.719	0.004	< 0.001	0.329
Playgrounds/sports fields 800 m				
≤1 (ref.)	1	1	1	1
2–5	0.87 (0.79–0.96)**	0.88 (0.80–0.97)**	1.16 (1.05–1.28)**	2.66 (2.35–3.00)**
6–10	0.96 (0.86–1.05)	0.96 (0.88–1.06)	1.24 (1.12–1.37)**	2.71 (2.39–3.06)**
≥11	0.90 (0.83–0.97)*	0.92 (0.85–0.99)*	1.42 (1.31–1.55)**	2.58 (2.35–2.84)**
P for trend	0.001	<0.001	<0.001	<0.001
Playgrounds/sports fields 5000 m				
≤35 (ref.)	1	1	1	1
36–119	0.99 (0.91–1.07)	0.98 (0.91–1.06)	0.98 (0.90–1.06)	1.68 (1.51–1.86)**
120–419	0.98 (0.90–1.06)	0.95 (0.88–1.03)	1.19 (1.09–1.29)**	1.69 (1.52–1.87)**
≥420	0.96 (0.88–1.04)	1.02 (0.95–1.10)	1.36 (1.25–1.47)**	1.51 (1.36–1.65)**
P for trend	0.079	0.953	<0.001	<0.001
School within 800 m				
No	1	1	1	1
Yes	0.94 (0.88–0.99)*	1.00 (0.94–1.06)	1.19 (1.11–1.28)**	1.16 (1.07–1.26)**
School within 5000 m				
No	1	1	1	1
Yes	0.94 (0.88–1.01)	0.98 (0.92–1.05)	1.27 (1.19–1.37)**	1.51 (1.39–1.64)**
Population density 800 m				
≤200 (ref.)	1	1	1	1
201–799	0.96 (0.88–1.03)	0.95 (0.88–1.03)	1.10 (1.02–1.20)**	2.18 (1.96–2.43)**
800–1649	0.96 (0.89–1.03)	0.99 (0.92–1.07)	1.30 (1.20–1.41)**	2.07 (1.87–2.30)**
≥1650	0.94 (0.87–1.02)	1.03 (0.96–1.11)	1.35 (1.24–1.47)**	1.56 (1.41–1.71)**
P for trend	0.735	0.041	<0.001	0.514

Note: OR, odds ratio; PA, physical activity. *p < 0.05. **p < 0.01

**Table 4:** Adjusted associations between environmental characteristics and activity participation in all children from MoBa

	All children (*n* = 21,309) Adjusted OR (95% CI)							
	≥ 5 h/week leisure-time PA (summer)		≥ 5 h/week leisure-time PA (winter)		Organized activities ≥2 days/week		Friends and peers ≥2 days/week	
	Step 1^a^	Step 2^b^	Step 1^a^	Step 2^b^	Step 1^a^	Step 2^b^	Step 1^a^	Step 2^b^
Total green space 800 m								
≤13% (ref.)	NI	NI	1	1	1	1	1	1
13.1–29.9%			1.15 (1.06–1.24)**	1.14 (1.05–1.23)**	1.02 (0.93–1.11)	1.02 (0.94–1.12)	0.87 (0.75–0.96)**	0.85 (0.76–0.95)**
30.0–49.9%			1.17 (1.08–1.26)**	1.15 (1.06–1.24)**	0.96 (0.88–1.04)	0.98 (0.90–1.07)	0.91 (0.82–1.02)	0.95 (0.85–1.07)
≥50.0%			1.27 (1.17–1.37)**	1.23 (1.13–1.33)**	0.89 (0.82–0.97)**	0.93 (0.85–1.02)	0.71 (0.64–0.79)**	0.83 (0.74–0.93)**
P for trend			< 0.001	0.002	0.164	0.813	< 0.001	0.001
Park within 800 m								
No (ref.)	1	1	NI	NI	1	1	NI	NI
Yes	1.02 (0.95–1.11)	1.12 (1.03–1.22)**			1.06 (0.97–1.15)	1.00 (0.91–1.09)		
Park within 5000 m								
No (ref.)	NI	NI	NI	NI	1	1	1	1
Yes					1.09 (1.02–1.16)**	1.01 (0.94–1.09)	1.53 (1.42–1.65)**	1.21 (1.10–1.34)**
Facilities/amenities 800 m								
0 (ref.)	NI	NI	NI	NI	1	1	1	1
1					1.17 (1.08–1.27)**	1.13 (1.04–1.22)**	1.33 (1.20–1.47)**	1.08 (0.97–1.20)
2–3					1.13 (1.05–1.23)**	1.09 (0.99–1.19)	1.43 (1.29–1.58)**	1.12 (1.00–1.26)
≥4					1.13 (1.03–1.25)**	1.08 (0.97–1.20)	1.19 (1.06–1.34)**	0.94 (0.82–1.07)
P for trend					0.009	0.205	0.214	0.005
Facilities/amenities 5000 m								
≤5 (ref.)	NI	NI	NI	NI	1	NE	1	NE
6–14					0.99 (0.91–1.08)		1.41 (1.27–1.57)**	
15–29					1.08 (0.99–1.18)		1.60 (1.43–1.78)**	
≥30					1.17 (1.07–1.27)**		1.49 (1.34–1.65)**	
P for trend					0.003		0.500	
Playgrounds/sports fields 800 m								
≤1 (ref.)	1	NE	1	NE	1	NE	1	NE
2–5	0.84 (0.76–0.93)**		0.84 (0.76–0.93)**		1.15 (1.04–1.27)**		2.78 (2.46–3.14)**	
6–10	0.90 (0.81–1.00)		0.89 (0.80–0.98)*		1.17 (1.05–1.30)**		2.91 (2.56–3.29)**	
≥11	0.80 (0.73–0.86)**		0.78 (0.72–0.85)**		1.29 (1.17–1.41)**		2.94 (2.67–3.24)**	
P for trend	< 0.001		< 0.001		< 0.001		< 0.001	
Playgrounds/sports fields 5000 m								
≤35 (ref.)	NI	NI	NI	NI	1	NE	1	NE
36–119					0.93 (0.86–1.02)		1.76 (1.59–1.95)**	
120–419					1.09 (0.99–1.19)		1.84 (1.66–2.04)**	
≥420					1.17 (1.07–1.28)**		1.76 (1.58–1.95)**	
P for trend					< 0.001		< 0.001	
School within 800 m								
No	1	1	NI	NI	1	1	1	1
Yes	0.89 (0.83–0.95)**	0.94 (0.88–1.01)			1.12 (1.05–1.20)**	1.08 (1.01–1.16)*	1.19 (1.10–1.30)**	0.99 (0.91–1.09)
School within 5000 m								
No	NI	NI	NI	NI	1	1	1	1
Yes					1.17 (1.09–1.26)**	1.11 (1.03–1.21)*	1.63 (1.49–1.77)**	1.19 (1.08–1.32)**
Population density 800 m								
≤200 (ref.)	NI	NI	NI	NI	1	–	1	–
201–799					1.05 (0.97–1.15)		2.28 (2.05–2.54)**	
800–1649					1.20 (1.10–1.30)**		2.25 (2.03–2.50)**	
≥1650					1.17 (1.07–1.27)**		1.80 (1.62–1.99)**	
P for trend					0.413		0.027	

Note: OR, odds ratio; PA, physical activity; NI, not included due to non-significance in bivariate models; NE, not estimated due to multicollinearity. *p < 0.05. ** p < 0.01

^a^Adjusted for sex, participation in after-school care, mother’s age and level of education

^b^Additional adjustment for population density

### Organized activities

Children with the greatest number of facilities/amenities and playgrounds/sports fields, both within a radius of 5000 m, had 17% higher odds of participating in organized activities ≥2 days/week. Playgrounds/sports fields within 800 m also supported participation, reaching 29% greater odds for children with ≥11 playgrounds/sports fields in their neighborhood (*p*_*trend*_ < 0.001). Furthermore, we found higher odds of participation for children who lived in more densely populated areas, with the greatest odds ratio for areas with moderate density (Table 4). In the individual- and area-level adjusted analyses, 8-year-olds with one facility within 800 m of their home had higher odds of participating in organized activities compared to children without any facilities (*p* < 0.01), but we did not observe a linear trend (Table 4). Access to school within 800 m was associated with 8% increased odds of participating in organized activities (*p* < 0.01).

### Informal social activities with friends/peers

Population density, facilities/amenities (5000-m radius) and playgrounds/sports fields (800- and 5000-m radii) remained statistically significantly associated with informal social activity with friends and peers ≥2 days/week after adjustment for individual-level covariates (Table 4). The associations were consistent across all quartiles, and the relation exhibited linear trends, except for facilities/amenities within 5000 m (*p*_*trend*_ = 0.500). The magnitude of the association was greatest for playgrounds/sports fields within 800 m. In the fully adjusted analyses, access to a park and school within 5000 m of home was related to 21 and 19% higher odds of participating in social activities ≥2 days/week, respectively. We did not identify any supportive associations for total neighborhood green space. Children who lived in neighborhoods classified as the lower (13.1–29.9% green space) and the upper (≥ 50.0% green space) quartiles had reduced odds of being together with friends and peers.

### Differences between boys and girls

Stratified analyses showed that associations varied between boys and girls (Tables 5, 6, and 7). Negative associations were observed between the number of playgrounds/sports fields (800-m) and leisure-time PA in the summer among boys, whereas playgrounds/sports fields (800- and 5000-m radii) and population density were negatively associated with leisure-time PA among girls. In the winter, these predictors were also related to decreased odds of ≥5 h/week leisure-time PA among girls. Greater proportions of total neighborhood green spaces were related to more leisure-time PA during the winter for both sexes. Several built environment exposures were positively related to boys’ participation in organized activities, but few characteristics supported such participation among girls. Population density, playgrounds/sports fields (800- and 5000-m radii) and facilities/amenities (5000-m radius) were associated with increased odds of social activity with friends and peers ≥2 days/week for both sexes.

**Table 5:** Crude associations between environmental characteristics and activity participation by gender

	Boys (*n* = 10,932) Crude OR (95% CI)				Girls (n = 10,214) Crude OR (95% CI)			
	≥ 5 h/week leisure-time PA (summer)	≥ 5 h/week leisure-time PA (winter)	Organized activities ≥2 days/week	Friends and peers ≥2 days/week	≥ 5 h/week leisure-time PA (summer)	≥ 5 h/week leisure-time PA (winter)	Organized activities ≥2 days/week	Friends and peers ≥2 days/week
Total green space 800 m								
≤13% (ref.)	1	1	1	1	1	1	1	1
13.1–29.9%	1.08 (0.96–1.21)	1.16 (1.04–1.30)**	1.01 (0.89–1.14)	0.84 (0.72–0.97)*	1.01 (0.91–1.13)	1.14 (1.02–1.27)*	1.03 (0.91–1.16)	0.89 (0.76–1.04)
30.0–49.9%	1.11 (0.99–1.25)	1.12 (1.01–1.25)*	0.91 (0.81–1.02)	0.90 (0.78–1.05)	1.05 (0.94–1.17)	1.15 (1.03–1.28)*	0.96 (0.85–1.09)	0.95 (0.81–1.12)
≥50.0%	1.07 (0.95–1.20)	1.18 (1.06–1.32)**	0.84 (0.75–0.94)**	0.71 (0.61–0.81)**	1.08 (0.96–1.21)	1.21 (1.08–1.35)**	0.87 (0.77–0.98)*	0.77 (0.67–0.89)**
P for trend	0.288	0.015	0.082	< 0.001	0.297	0.045	0.117	0.001
Park within 800 m								
No (ref.)	1	1	1	1	1	1	1	1
Yes	1.07 (0.96–1.19)	1.04 (0.94–1.15)	1.14 (1.02–1.28)*	1.06 (0.92–1.22)	1.10 (0.99–1.22)	1.06 (0.95–1.17)	1.11 (0.98–1.25)	1.04 (0.90–1.21)
Park within 5000 m								
No (ref.)	1	1	1	1	1	1	1	1
Yes	1.00 (0.92–1.09)	1.08 (0.99–1.16)	1.27 (1.17–1.38)**	1.41 (1.28–1.57)**	0.97 (0.89–1.05)	1.01 (0.93–1.09)	1.10 (1.01–1.21)*	1.42 (1.27–1.59)**
Facilities/amenities 800 m								
0 (ref.)	1	1	1	1	1	1	1	1
1	0.98 (0.88–1.09)	1.07 (0.97–1.18)	1.20 (1.08–1.34)**	1.28 (1.11–1.46)**	0.90 (0.81–1.00)	0.93 (0.84–1.04)	1.19 (1.06–1.34)**	1.33 (1.14–1.54)**
2–3	1.01 (0.91–1.13)	1.05 (0.94–1.16)	1.24 (1.11–1.39)**	1.37 (1.19–1.58)**	0.96 (0.86–1.06)	0.95 (0.86–1.06)	1.14 (1.01–1.28)*	1.38 (1.18–1.60)**
≥4	0.97 (0.85–1.10)	1.07 (0.95–1.21)	1.21 (1.06–1.37)**	1.13 (0.97–1.33)	0.93 (0.82–1.05)	0.93 (0.82–1.05)	1.29 (1.12–1.48)**	1.12 (0.95–1.32)
P for trend	0.912	0.041	0.005	0.976	0.254	0.503	0.001	0.441
Facilities/amenities 5000 m								
≤5 (ref.)	1	1	1	1	1	1	1	1
6–14	1.04 (0.93–1.17)	1.08 (0.97–1.20)	1.14 (1.02–1.28)*	1.36 (1.18–1.57)**	1.10 (0.99–1.23)	1.02 (0.91–1.14)	0.94 (0.83–1.06)	1.33 (1.15–1.55)**
15–29	1.08 (0.97–1.22)	1.11 (1.00–1.24)	1.27 (1.13–1.42)**	1.68 (1.45–1.95)**	0.95 (0.85–1.07)	0.95 (0.85–1.06)	1.04 (0.92–1.18)	1.31 (1.12–1.52)**
≥30	1.03 (0.92–1.15)	1.16 (1.04–1.28)**	1.45 (1.30–1.62)**	1.31 (1.14–1.49)**	0.95 (0.86–1.06)	0.99 (0.89–1.10)	1.23 (1.09–1.38)**	1.33 (1.15–1.54)**
P for trend	0.288	< 0.001	< 0.001	0.107	0.232	0.589	< 0.001	0.815
Playgrounds/sports fields 800 m								
≤1 (ref.)	1	1	1	1	1	1	1	1
2–5	0.86 (0.75–0.99)*	0.90 (0.79–1.02)	1.25 (1.09–1.44)**	2.81 (2.38–3.32)**	0.88 (0.77–1.01)	0.86 (0.75–0.99)*	1.06 (0.92–1.23)	2.49 (2.09–2.97)**
6–10	0.98 (0.85–1.13)	1.00 (0.88–1.15)	1.29 (1.12–1.48)**	2.75 (2.32–3.26)**	0.95 (0.83–1.09)	0.94 (0.82–1.08)	1.18 (1.01–1.37)*	2.64 (2.20–3.17)**
≥11	0.97 (0.87–1.10)	1.01 (0.90–1.12)	1.54 (1.37–1.72)**	2.66 (2.35–3.02)**	0.84 (0.75–0.94)**	0.85 (0.76–0.95)**	1.30 (1.15–1.47)**	2.49 (2.17–2.85)**
P for trend	0.529	0.052	< 0.001	< 0.001	< 0.001	< 0.001	< 0.001	< 0.001
Playgrounds/sports fields 5000 m								
≤35 (ref.)	1	1	1	1	1	1	1	1
36–119	1.08 (0.96–1.21)	1.08 (0.97–1.20)	1.00 (0.89–1.12)	1.56 (1.35–1.79)**	0.93 (0.83–1.04)	0.90 (0.81–1.01)	0.95 (0.84–1.07)	1.81 (1.56–2.11)**
120–419	1.05 (0.94–1.18)	1.02 (0.91–1.13)	1.24 (1.11–1.40)**	1.86 (1.61–2.14)**	0.91 (0.81–1.01)	0.88 (0.79–0.99)*	1.12 (0.99–1.27)	1.52 (1.31–1.76)**
≥420	1.04 (0.93–1.16)	1.12 (1.01–1.24)*	1.46 (1.30–1.64)**	1.42 (1.24–1.62)**	0.90 (0.81–1.00)	0.94 (0.84–1.05)	1.24 (1.10–1.41)**	1.62 (1.40–1.88)**
P for trend	0.603	0.262	< 0.001	0.006	0.059	0.311	< 0.001	< 0.001
School within 800 m								
No	1	1	1	1	1	1	1	1
Yes	0.96 (0.88–1.05)	1.06 (0.98–1.15)	1.23 (1.12–1.35)**	1.14 (1.02–1.28)*	0.92 (0.84–1.00)	0.95 (0.87–1.04)	1.15 (1.04–1.27)**	1.18 (1.05–1.34)**
School within 5000 m								
No	1	1	1	1	1	1	1	1
Yes	1.00 (0.91–1.11)	1.09 (0.99–1.19)	1.33 (1.21–1.47)**	1.37 (1.23–1.54)**	0.90 (0.82–0.99)*	0.88 (0.80–0.97)*	1.21 (1.09–1.34)**	1.67 (1.48–1.89)**
Population density 800 m								
≤200 (ref.)	1	1	1	1	1	1	1	1
201–799	1.03 (0.92–1.16)	1.05 (0.94–1.17)	1.16 (1.03–1.30)**	2.15 (1.86–2.48)**	0.92 (0.82–1.03)	0.88 (0.79–0.98)*	1.03 (0.92–1.17)	2.21 (1.89–2.59)**
800–1649	1.05 (0.94–1.17)	1.06 (0.95–1.18)	1.35 (1.20–1.51)**	2.27 (1.97–2.62)**	0.89 (0.80–0.99)*	0.94 (0.84–1.05)	1.25 (1.10–1.41)**	1.87 (1.61–2.17)**
≥1650	1.06 (0.94–1.18)	1.15 (1.03–1.28)**	1.46 (1.30–1.64)**	1.48 (1.30–1.69)**	0.87 (0.78–0.97)**	0.94 (0.84–1.05)	1.23 (1.09–1.39)**	1.64 (1.42–1.90)**
P for trend	0.064	0.003	<0.001	0.381	0.273	0.942	0.101	0.065

Note: OR, odds ratio; PA, physical activity. *p < 0.05. **p < 0.01

**Table 6:** Adjusted associations between environmental characteristics and activity participation in boys from MoBa

	Adjusted OR (95% CI) in boys (*n* = 10,932)							
	≥ 5 h/week leisure-time PA (summer)		≥ 5 h/week leisure-time PA (winter)		Organized activities ≥2 days/week		Friends and peers ≥2 days/week	
	Step 1^a^	Step 2^b^	Step 1^a^	Step 2^b^	Step1^a^	Step 2^b^	Step 1^a^	Step 2^b^
Total green space 800 m								
≤13% (ref.)	NI	NI	1	1	1	1	1	1
13.1–29.9%			1.16 (1.04–1.29)*	1.16 (1.03–1.29)*	1.01 (0.90–1.14)	1.03 (0.91–1.16)	0.84 (0.73–0.98)*	0.82 (0.71–0.95)*
30.0–49.9%			1.15 (1.04–1.28)**	1.15 (1.03–1.28)*	0.93 (0.83–1.04)	0.97 (0.86–1.10)	0.89 (0.77–1.03)	0.92 (0.79–1.07)
≥50.0%			1.26 (1.13–1.40)**	1.25 (1.11–1.40)**	0.87 (0.78–0.98)*	0.95 (0.84–1.08)	0.69 (0.59–0.79)**	0.80 (0.68–0.93)**
P for trend			0.001	0.004	0.237	0.823	<0.001	0.002
Park within 800 m								
No (ref.)	NI	NI	NI	NI	1	1	NI	NI
Yes					1.09 (0.97–1.22)	0.99 (0.87–1.12)		
Park within 5000 m								
No (ref.)	NI	NI	NI	NI	1	1	1	1
Yes					1.18 (1.09–1.29)**	1.07 (0.97–1.19)	1.54 (1.37–1.71)**	1.20 (1.05–1.37)**
Facilities/amenities 800 m								
0 (ref.)	NI	NI	NI	NI	1	1	1	1
1					1.17 (1.05–1.31)**	1.11 (0.99–1.24)	1.31 (1.14–1.50)**	1.06 (0.92–1.22)
2–3					1.19 (1.07–1.34)**	1.09 (0.97–1.24)	1.44 (1.25–1.66)**	1.14 (0.97–1.33)
≥4					1.11 (0.97–1.27)	0.99 (0.86–1.15)	1.21 (1.03–1.42)**	0.96 (0.80–1.15)
P for trend					0.089	0.968	0.503	0.022
Facilities/amenities 5000 m								
≤5 (ref.)	NI	NI	1	NE	1	NE	1	NE
6–14			0.99 (0.90–1.11)		1.10 (0.98–1.24)		1.44 (1.25–1.65)**	
15–29			0.99 (0.88–1.10)		1.20 (1.06–1.35)**		1.82 (1.57–2.11)**	
≥30			0.93 (0.83–1.04)		1.30 (1.16–1.47)**		1.51 (1.31–1.74)**	
P for trend			0.862		0.001		0.920	
Playgrounds/sports fields 800 m								
≤1 (ref.)	1	NE	NI	NE	1	NE	1	NE
2–5	0.84 (0.73–0.96)*				1.24 (1.08–1.43)**		2.97 (2.52–3.52)**	
6–10	0.92 (0.80–1.06)				1.23 (1.06–1.42)**		2.98 (2.51–3.54)**	
≥11	0.87 (0.77–0.98)*				1.42 (1.26–1.60)**		3.09 (2.70–3.52)**	
P for trend	<0.001				<0.001		<0.001	
Playgrounds/sports fields 5000 m								
≤35 (ref.)	NI	NI	1	NE	1	NE	1	NE
36–119			1.00 (0.89–1.11)		0.96 (0.86–1.08)		1.65 (1.43–1.90)**	
120–419			0.89 (0.79–0.99)*		1.16 (1.03–1.31)*		2.04 (1.77–2.37)**	
≥420			0.88 (0.78–0.98)*		1.30 (1.15–1.47)**		1.68 (1.45–1.94)**	
P for trend			0.004		<0.001		<0.001	
School within 800 m								
No	NI	NI	NI	NI	1	1	1	1
Yes					1.18 (1.07–1.29)**	1.09 (0.99–1.21)	1.18 (1.06–1.33)**	0.98 (0.86–1.11)
School within 5000 m								
No	NI	NI	NI	NI	1	1	1	1
Yes					1.24 (1.13–1.37)**	1.14 (1.02–1.28)*	1.50 (1.33–1.69)**	1.05 (0.92–1.21)
Population density 800 m								
≤200 (ref.)	NI	–	1		1	–	1	–
201–799			0.98 (0.88–1.09)		1.11 (0.99–1.25)		2.26 (1.94–2.61)**	
800–1649			0.94 (0.84–1.04)		1.27 (1.13–1.52)**		2.49 (2.15–2.88)**	
≥1650			0.91 (0.82–1.02)		1.30 (1.15–1.47)**		1.74 (1.52–2.01)**	
P for trend			0.537		0.031		0.617	

Note: OR, odds ratio; PA, physical activity; NI, not included due to non-significance in bivariate models; NE, not estimated due to multicollinearity. *p < 0.05. ** *p* < 0.01

^a^Adjusted for participation in after-school care, mother’s age and level of education

^b^Additional adjustment for population density

**Table 7:** Adjusted associations between environmental characteristics and activity participation in girls from MoBa

	Adjusted OR (95% CI) in girls (n = 10,214)							
	≥ 5 h/week leisure-time PA (summer)		≥ 5 h/week leisure-time PA (winter)		Organized activities ≥2 days/week		Friends and peers ≥2 days/week	
	Step 1^a^	Step 2^b^	Step 1^a^	Step 2^b^	Step 1^a^	Step 2^b^	Step 1^a^	Step 2^b^
Total green space 800 m								
≤13% (ref.)	NI	NI	1	1	1	1	1	1
13.1–29.9%			1.14 (1.02–1.27)*	1.13 (1.01–1.26)*	1.03 (0.91–1.17)	1.02 (0.90–1.16)	0.89 (0.76–1.04)	0.88 (0.75–1.04)
30.0–49.9%			1.19 (1.06–1.33)**	1.15 (1.02–1.29)*	1.00 (0.88–1.13)	0.99 (0.87–1.13)	0.94 (0.80–1.10)	1.00 (0.85–1.18)
≥50.0%			1.28 (1.15–1.44)**	1.20 (1.07–1.36)**	0.91 (0.81–1.04)	0.92 (0.80–1.05)	0.75 (0.64–0.88)**	0.88 (0.75–1.04)
P for trend			0.005	0.115	0.456	0.604	<0.001	0.108
Park within 800 m								
No (ref.)	NI	NI	NI	NI	NI	NI	NI	NI
Yes								
Park within 5000 m								
No (ref.)	NI	NI	NI	NI	1	1	1	1
Yes					0.99 (0.90–1.09)	0.94 (0.84–1.05)	1.51 (1.35–1.69)**	1.23 (1.07–1.41)**
Facilities/amenities 800 m								
0 (ref.)	NI	NI	NI	NI	1	1	1	1
1					1.16 (1.03–1.31)*	1.17 (1.03–1.32)*	1.35 (1.17–1.57)**	1.10 (0.94–1.29)
2–3					1.07 (0.95–1.20)	1.08 (0.95–1.23)	1.41 (1.21–1.64)**	1.11 (0.94–1.31)
≥4					1.16 (1.01–1.33)*	1.17 (1.01–1.37)*	1.17 (0.99–1.38)	0.91 (0.76–1.10)
P for trend					0.047	0.066	0.270	0.089
Facilities/amenities 5000 m								
≤5 (ref.)	NI	NI	NI	NI	1	NE	1	NE
6–14					0.88 (0.78–1.00)		1.38 (1.19–1.61)**	
15–29					0.96 (0.84–1.09)		1.38 (1.18–1.61)**	
≥30					1.03 (0.90–1.16)		1.47 (1.26–1.71)**	
P for trend					0.383		0.280	
Playgrounds/sports fields 800 m								
≤1 (ref.)	1	NE	1	NE	1	NE	1	NE
2–5	0.85 (0.74–0.97)*		0.83 (0.72–0.95)**		1.04 (0.90–1.21)		2.57 (2.15–3.07)**	
6–10	0.88 (0.76–1.01)		0.85 (0.74–0.98)*		1.10 (0.94–1.28)		2.82 (2.34–3.39)**	
≥11	0.73 (0.65–0.82)**		0.72 (0.64–0.80)**		1.15 (1.01–1.31)*		2.78 (2.41–3.21)**	
P for trend	<0.001		<0.001		0.061		<0.001	
Playgrounds/sports fields 5000 m								
≤35 (ref.)	NI	NE	1	NE	1	NE	1	NE
36–119			0.84 (0.75–0.94)**		0.90 (0.80–1.02)		1.89 (1.63–2.21)**	
120–419			0.77 (0.68–0.86)**		1.01 (0.89–1.15)		1.63 (1.41–1.90)**	
≥420			0.74 (0.66–0.83)**		1.04 (0.91–1.18)		1.85 (1.58–2.16)**	
P for trend			<0.001		0.289		<0.001	
School within 800 m								
No	NI	NI	NI	NI	1	1	1	1
Yes					1.07 (0.97–1.18)	1.06 (0.95–1.18)	1.21 (1.07–1.37)**	1.01 (0.88–1.16)
School within 5000 m								
No	1	1	1	1	1	1	1	1
Yes	0.81 (0.74–0.90)**	0.91 (0.82–1.02)	0.78 (0.70–0.86)**	0.84 (0.75–0.94)**	1.09 (0.98–1.22)	1.08 (0.96–1.23)	1.79 (1.58–2.03)**	1.38 (1.19–1.59)**
Population density 800 m								
≤200 (ref.)	1	–	1		1	–	1	–
201–799	0.87 (0.77–0.97)*		0.82 (0.74–0.92)**		0.99 (0.87–1.11)		2.30 (1.97–2.69)**	
800–1649	0.79 (0.70–0.88)**		0.82 (0.73–0.92)**		1.12 (0.98–1.27)		2.01 (1.73–2.34)**	
≥1650	0.71 (0.63–0.80)**		0.75 (0.67–0.84)**		1.03 (0.91–1.18)		1.86 (1.60–2.17)**	
P for trend	<0.001		0.002		0.275		0.008	

Note: OR, odds ratio; PA, physical activity; NI, not included due to non-significance in bivariate models; NE, not estimated due to multicollinearity. *p < 0.05. ** *p* < 0.01

^a^Adjusted for participation in after-school care, mother’s age and level of education

^b^Additional adjustment for population density

### Results from the sensitivity analysis

Additional file 2: Table S1 presents the results from the sensitivity analysis. In general, the pattern and the magnitude of the estimated ORs across all outcomes were consistent with the main results shown in Table 4, although several significant associations vanished (mainly for organized activities). The positive significant associations observed between the built environment and participation in social activity with friends remained significant and strong, or were even slightly stronger, in the sub sample of children who participated in the 8-year follow-up in 2014 and 2015.

## Discussion

### Main findings

This study showed that children with access to a park in their neighborhood were more physically active during the summer than those without such access. Moreover, children who lived in neighborhoods with higher proportions of green space participated more in PA during the winter than children who lived in neighborhoods with low proportions of green space. More densely populated areas and access to facilities such as playgrounds/sports fields and schools were related to participation in organized activities and social activities. A higher number of playgrounds/sports fields in the neighborhood was the strongest correlate of leisure-activities in the Norwegian 8-year-olds, which consistently was linked more socialization with friends. We also found differential associations by sex. Several built environment characteristics were negatively related to leisure-time PA in the summer and the winter among girls but not among boys. Further, there were few supportive associations between the built environment and girls’ participation in organized activities. More playgrounds/sports fields in the neighborhood was strongly related to social activity with friend and peers among both boys and girls.

### The built environment and leisure-time PA

The findings of neighborhood green spaces as potential supportive predictors of leisure-time PA in children agree with previously reported results [24, 47]. However, several studies did not support favorable associations between access to green spaces and PA [48, 49]. These inconsistencies in results across studies could partly be attributed to the heterogeneity between studies with respect to methodology applied and how the built environment characteristics are operationalized [50], and our results add to this body of equivocal literature [16, 19, 50]. Interestingly, we observed that associations between neighborhood green spaces and leisure-time PA were somewhat more pronounced in the winter than in the summer. The Norwegian climate is generally characterized by large seasonal variations with relatively warm summers and cold winters with snow in parts of the season. Furthermore, Norway is a country with strong outdoor traditions throughout the year. The majority of the population, including children, spend time outdoors almost regardless of the weather [51, 52]. These seasonal variations and cultural factors, which allow children to engage in a broad range of outdoor activities, can explain the results. It is highly conceivable that neighborhood parks serve as venues for summer activities (like ball games, biking, and running), whereas in the winter, neighborhood green spaces (like forests, marshland, and other open areas) afford more opportunities for common activities such as skiing and tobogganing.

We found that access to playgrounds/sports fields and schools was associated with reduced odds of PA among 8-year-olds, particularly for girls. These results diverge from what is widely accepted for the general population [14], but they agree with a meta-analysis of GIS studies that identified negative relations between access to play space and facilities among children [19]. Parental concerns and restrictions are the prevailing explanations for these results [19, 53]. In particular, concerns about traffic safety are reported as common reasons why parents restrict children from using their neighborhood surroundings [54, 55]. Generally, neighborhood areas with higher density have more facilities, and traffic congestion increases with population density [31, 32]. Thus, parental concerns for young children’s safety may deter parents from allowing their children to be outside, especially if there are many traffic-related barriers. Even if opportunities for activities are present near home, they might not be reached or be used for other reasons, which unfortunately, we were not able to consider in this study. This explanation likely applies to the present study and to parents of Norwegian 8-year-olds. More research revealing the processes and mechanisms underlying these relations is needed. Future studies should consider other interpersonal factors (e.g., parental perceptions of the environment and activity preferences, as well as family-level characteristics, such as having older siblings) that could have an impact on younger children’s opportunities to take part in leisure activities.

Another aspect that can shed light on these results is that the majority (78.0%) of the Norwegian population lives in detached houses, duplexes, or terraced houses, of which detached houses are most common (56.6%) [56]. Thus, Norwegian children likely spend a great amount of their leisure-time PA in their private gardens or backyards. The participants did not provide information about housing, but we assume that many children in this sample have access to private spaces that provide opportunities for PA. This can also explain why neighborhood facilities were less and even negatively related to children’s PA. Access to gardens and backyards could be important predictors of PA among children in Norway and warrant more attention.

### The built environment and participation in organized activities

We did not measured participation in specific activities, but the survey questions considered organized activities broadly. Thus, the results provide some novel insights by showing that facilities, playgrounds/sports fields and school within 800- and 5000 m of children’s home were related to participation in organized activities among Norwegian 8-year-olds. Few studies have investigated relations between the built environment and children’s participation in organized activities using GIS-derived measures. The existing studies mainly considered organized sports [23, 57]. Neither Buck et al. [23] nor Galvez et al. [57] reported statistically significant associations between access to facilities and organized sports among children.

Samdal and coworkers [58] reported that Norwegian adolescents engage most often in team or individual sports. This finding likely applies to children as well. In Norway, schools are important community arenas, and team and individual sports (e.g., handball, soccer, dancing, and martial arts) commonly take place at schools. It is highly conceivable that the school is the most relevant venue for team and individual sports, which could explain the present finding. Likewise, we observed that access to more facilities was positively related to engagement in both organized and social activities. A recent study revealed that children find their meaningful places for activities in both educational, commercial, recreational, traffic and religious behavior settings [59]. The total facility measure used in the present study included activity venues such as indoor pools, churches, shopping malls and community centers. Assuming that more facilities are linked to a greater mix of facilities, our results suggest that many facilities could be essential for meeting children’s different activity preferences and thus, support participation.

### The built environment and socialization with friends/peers

Only a handful of studies have examined the relation between the built environment and social activity with friends and peers among children [20]. A study of U.S. children showed that living in neighborhoods with poor physical conditions and few facilities is linked to less time spent in peer play [60]. We add to this limited evidence by unveiling that higher population density and greater access to facilities, as well as park areas, promote participation in social activity with friends. Across all built characteristics, access to playgrounds/sports fields within 800 m of the child’s residence was the strongest correlate of socialization with friends and peers. Mouratidis [61] recently reported similar results showing that shorter distances to facilities and higher population density facilitate more frequent socializing among Norwegian adults.

Unlike access to facilities, total neighborhood green space decreased the odds of social activity with friends and peers. This result is likely underpinned by the fact that greener areas are less dense. We found the highest likelihood of participating in organized activities for children in neighborhoods with moderate population density. Moreover, we observed that children living in densely populated areas had higher odds of spending time with friends compared to children who did not lived in densely populated neighborhoods, and the associations were consistent across all quartiles. This finding indicates that living in densely populated neighborhoods increases children’s possibilities to meet friends. However, greatest likelihood of engagement in social activity was observed for children in the quartile described as lower degrees of density closely followed by moderate degrees of density. As such, one might question which degrees of density that have the strongest potential to promote participation in leisure-activities. In Finland, researchers have shown that moderate urban density has child-friendly characteristics, such as ensuring shorter distances to meaningful places [62]. Based on this study and the increased centralization in settlement patterns in Norway [63], the role of density in creating health-promoting and supportive childhood environments should be further explored within the Norwegian context.

### The neighborhood and the larger community

Built environment exposures within the 800- and 5000-m radii were associated with participation in organized and social activity, whereas only exposures within the 800-m radius were relevant for PA. One reason for the observed difference could be that parents are more involved in children’s organized and social activities, while leisure-time PA is more self-governed. Dunton et al. [64] provided support for the importance of parents’ presence showing that children’s leisure-activities often occur with family members. Traffic safety and other parental restrictions and concerns are significantly less prominent when parents accompany their children to activities. Thus, parents expand children’s spatial territory, which can explain why other predictors and larger spatial areas were related to participation in organized and social activities with friends and peers. Interestingly, Kenney [60] found that parent-perceived neighborhood safety was not linked to peer play. Children might be allowed to roam more and actualize affordances when they are with friends and peers, which provides support for the present results.

### Strengths and limitations

The strengths of this study lie in the large sample of 8-year-olds from across Norway linked to rich environmental data about the built environment. This large sample provided a unique opportunity to investigate associations between the built environment and participation in leisure activities in childhood. Unlike previous research, we were able to study participation in organized and social activities, as well as examine leisure-time PA across seasons. Although there is lack of consensus about how to define the areas of exposure and built environment predictors of interest, we operationalized the GIS measures based on previous empirical work of measures applied among children and adolescents [37]. Use of objectively measured exposures also eliminated the potential risk of single source bias.

We could not infer causal relations from this cross-sectional design. Although we identified and adjusted for the most important confounders, other variables not included in the MoBa, could confound the associations between the built environment and activity outcomes. Environmental variables not measured, such as traffic exposure and safety aspects, could also confound the results. Furthermore, the results are vulnerable to residential self-selection bias stemming from the non-random selection of children into neighborhoods based on their parents’ preferences [65]. Young women and mothers living alone were underrepresented in the MoBa [66]. Additionally, children of younger and lower-educated mothers were less likely to be included in our analyses, which also increased the risk of selection and attrition bias. However, Nilsen et al. [66], who compared participants in MoBa with the Medical Birth Registry in Norway, identified little bias in other exposure–outcome associations, indicating that selection bias may not be a serious problem in studies such as the present study.

Considering misclassification, we were not able to compute a child’s actual exposure to the built environment and used buffer zones around residences as proxies. To reduce the likelihood of error, we excluded children living in post-separation families to make certain that the child lived at the actual address used for the calculation. The children participated in the 8-year follow-up between 2011 and 2015, whereas we obtained GIS-data from 2016 and January 2017 only. Thus, we did not establish the temporal sequencing from exposure to outcome. New parks, playgrounds, and facilities may have been developed, which potentially could have led to misclassification of exposures, and the risk of misclassification is highest for children participating in 2011. We are aware of these issues, but the built environment is postulated to transform slowly [67]. As such, large infrastructural changes to the built environment between 2011 and 2016 is less likely. Further, the risk of error due to changes in the built environment is expected to be minor for children followed-up in 2014 and 2015. In support of these notions, the sensitivity analysis in the sub-sample of children participating in 2014 and 2015 showed virtually equal ORs. That several significant results disappeared could be explained by a lower number of participants reducing the power to detect smaller differences. Thus, the results seem to be less susceptible to information bias, but if such bias are present, we suggest that the exposure most likely is non-differentially misclassified.

We did not measure the use and quality of the facilities and green spaces. It must be acknowledged that factors other than provision, such as safety and aesthetics, might influence the actualization of affordances [14]. Although we did not conduct a formal assessment, it was evident while we completed the GIS measures that many of the playgrounds were small and had limited space for activities such as running. Moreover, although separate measures for schools and playgrounds/sports fields were computed, we did not differentiate between various types of facilities when we assessed the total number of facilities. As the literature suggests [37], qualities of green spaces and other venues for activities should be more extensively studied among children in Norway, as well as elsewhere, and more specific measures of different types of facilities should be applied. Further, the GIS measures were calculated using vector-based geographical data. We ran into computational challenges and were not able to calculate a child’s exposure to population density and total green space within the 5000-m radius. Raster-based data could have resolved our computational challenges, but unfortunately, such data were not obtained. Additionally, we relied on parental-reported data on children’s leisure activities. This could be considered a limitation as such measures are susceptible to both recall bias and social desirability bias.

Lastly, the results specifically apply to the Norwegian context. Norway is characterized by rich access to green space and low population density [68]. Moreover, the seasonal variations in weather as well as other social and cultural factors, such as our strong outdoor traditions and differences in parenting norms, must be considered. Inevitably, the study findings may not be widely generalizable to other countries. However, it is still reasonable to assume that some of the findings could be applicable to other Scandinavian countries where certain similarities in contextual, social and cultural factors are present.

## Conclusion and implications

This first Norwegian population-based study using GIS-derived measures of the built environment provides confirmatory and novel empirical evidence of the built environment characteristics that promote activity participation in childhood. Green spaces, facilities, playgrounds/sports fields, and population density supported participation in leisure-activities. Each built environment characteristic likely provides opportunities for different activities that are important for children’s health and well-being. Although the results should be interpreted with caution, they underscore the importance of having access to a variety of venues and affordances for different activities in the immediate neighborhood surroundings, as well as in the greater community. These findings convey some suggestions that can inform health-promoting strategies and planning decisions to secure future development of neighborhoods that are inclusive for all. In particular, the present results point to the importance of providing access to playgrounds/sports fields in existing neighborhoods as well as areas under development. Furthermore, attention to and consideration of the design of green spaces to accommodate for seasonal activities should be given in planning and development processes. This has the capacity to enhance children’s well-being and public health in general. This study also elucidates that creating health-promoting environments is complex and not straightforward, which points to the importance of integrative planning practices and solutions, as well as closer collaboration among researchers, policy makers, planners, and public health professionals.

## Supplementary information

**Additional file 1: Figure S1.** A directed acyclic graph depicting the relations between exposures, outcomes and potential covariates. The figure shows a directed acyclic graph (DAG) depicting the links between exposures, outcomes and covariates. Through the DAG, we identified which confounders to include in the statistical analyses to sufficiently control for potential confounders.

**Additional file 2: Table S1.** Sensitivity analysis of selected children who participated in the 8-year follow-up in 2014 and 2015. The table presents the adjusted results from the sensitivity analysis conducted in the sub sample of 8311 children.

## Data Availability

The data that support the findings of this study are available from the Norwegian Institute of Public Health but restrictions apply to the availability due to agreements and approvals involving data security for participants from the Norwegian Data Protection Authority, Regional Committee for Medical Research Ethics as well as regulations in the Norwegian Health Registry Act. Data are however available from the authors upon reasonable request and with permission of the data owner if requestors wish to access the data for the purposes of checking analyses.

## Abbreviations

CI: confidence interval
GIS: geographic information systems
MoBa: The Norwegian Mother and Child Cohort Study
OR: odds ratio
PA: physical activity
VIF: variance inflation factor
