# Supplementary material for: Neighborhood green spaces, facilities and population density as predictors of activity participation among 8-year-olds: a cross-sectional GIS study based on the Norwegian mother and child cohort study
Source: BMC Public Health. 2019 Oct 30;19:1426. doi: 10.1186/s12889-019-7795-9 (PMC6822450; doi:10.1186/s12889-019-7795-9)
Supplement: Supplementary file 1 — Additional file 1: Figure S1. A directed acyclic graph depicting the relations between exposures, outcomes and potential covariates. The figure shows a directed acyclic graph (DAG) depicting the links between exposures, outcomes and covariates. Through the DAG, we identified which confounders to include in the statistical analyses to sufficiently control for potential confounders. [file 12889_2019_7795_MOESM1_ESM.docx]

**Additional file 1**

**Neighborhood Green Spaces, Facilities and Population Density as Predictors of Activity Participation among 8-Year-Olds: A Cross-Sectional GIS study Based on the Norwegian Mother and Child Cohort Study**

Emma Charlott Andersson Nordbø,^*1,2^ Ruth Kjærsti Raanaas,^1,2^ Helena Nordh,^1^ and Geir Aamodt^1^

^1^ Department of Public Health Science, Faculty of Landscape and Society, Norwegian University of Life Sciences, Ås, Norway

^2^ The Centre for Evidence-Based Public Health: A Joanna Briggs Institutes Affiliated Group

**Table of Contents**

**Figure S1.** Directed acyclic graph depicting the relations between the exposures, outcomes and potential covariates.


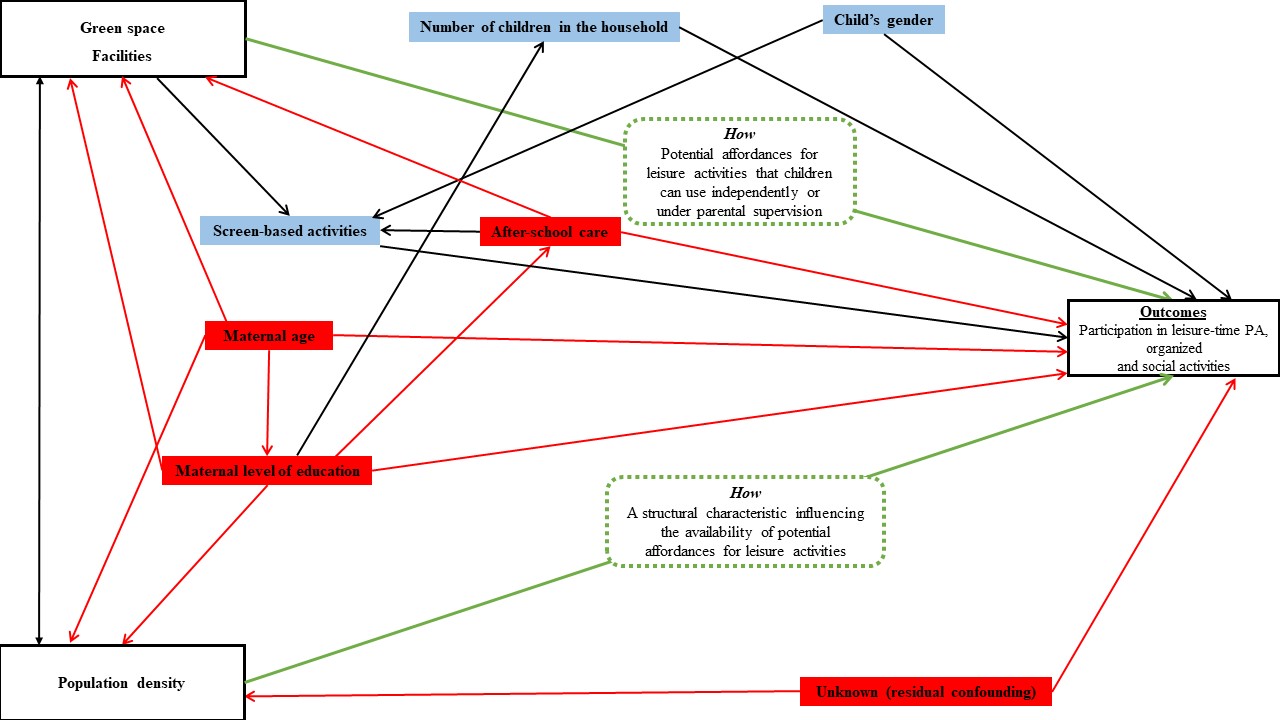


Note: The read arrows and boxes represent confounded paths and the related confounding variables. The black arrows are unconfounded paths. The blue boxes represent covariates that are related to both the exposures and the outcomes, but not considered as confounders. The green dotted boxes describe how the built environment exposures might relate to children’s participation in leisure activities. To estimate the overall association between the built environment exposures and the activity outcomes minimal sufficient adjustment were identified to include the following confounders and covariates: child’s gender, maternal age and level of education, after-school care and population density.

**Figure S1.** Directed acyclic graph depicting the relations between the exposures, outcomes and potential covariates.
