# Supplementary material for: Neighborhood green spaces, facilities and population density as predictors of activity participation among 8-year-olds: a cross-sectional GIS study based on the Norwegian mother and child cohort study
Source: BMC Public Health. 2019 Oct 30;19:1426. doi: 10.1186/s12889-019-7795-9 (PMC6822450; doi:10.1186/s12889-019-7795-9)
Supplement: Supplementary file 2 — Additional file 2: Table S1. Sensitivity analysis of selected children who participated in the 8-year follow-up in 2014 and 2015. The table presents the adjusted results from the sensitivity analysis conducted in the sub sample of 8311 children. [file 12889_2019_7795_MOESM2_ESM.docx]

**Additional file 2**

**Neighborhood Green Spaces, Facilities and Population Density as Predictors of Activity Participation among 8-Year-Olds: A Cross-Sectional GIS study Based on the Norwegian Mother and Child Cohort Study**

Emma Charlott Andersson Nordbø,^*1,2^ Ruth Kjærsti Raanaas,^1,2^ Helena Nordh,^1^ and Geir Aamodt^1^

^1^ Department of Public Health Science, Faculty of Landscape and Society, Norwegian University of Life Sciences, Ås, Norway

^2^ The Centre for Evidence-Based Public Health: A Joanna Briggs Institutes Affiliated Group

**Table of Contents**

**Table S1.** Sensitivity analysis of selected children who participated in the 8-year follow-up in 2014 and 2015.

**Table S1.** Sensitivity analysis of selected children who participated in the 8-year follow-up in 2014 and 2015.

|  | Selected children (N = 8 311) Adjusted OR (95 % CI) | | | | | | | |
| --- | --- | --- | --- | --- | --- | --- | --- | --- |
|  | > 5 h/week leisure-time PA (summer)  Step 1^a^ Step 2^b^ | | > 5 h/week leisure-time PA (winter)  Step 1^a^  Step 2^b^ | | Organized activities > 2 days/week  Step 1^a^ Step 2^b^ | | Friends and peers > 2 days/week  Step 1^a^ Step 2^b^ | |
| Total green space 800 m  < 13 % (ref.)  13.1 – 29.9 %  30.0 – 49.9 %  > 50.0 %  P for trend | NI | NI | 1  1.19 (1.06–1.35)**  1.11 (0.98–1.26)  1.31 (1.16–1.49)**  <0.001 | 1  1.18 (1.04–1.33)**  1.08 (0.95–1.23)  1.26 (1.10–1.44)**  0.017 | 1  1.00 (0.87–1.15)  0.93 (0.81–1.07)  0.92 (0.80–1.06)  0.916 | 1  0.99 (0.86–1.13)  0.91 (0.79–1.05)  0.89 (0.77–1.04)  0.792 | 1  0.90 (0.77–1.06)  0.94 (0.80–1.11)  0.77 (0.66–0.91)**  0.001 | 1  0.89 (0.76–1.05)  1.00 (0.84–1.19)  0.84 (0.79–1.12)  0.241 |
| Park within 800 m  No (ref.)  Yes | 1  1.01 (0.90–1.15) | 1  1.09 (0.96–1.23) | NI | NI | 1  1.01 (0.89–1.14) | 1  1.02 (0.90–1.17) | NI | NI |
| Park within 5 000 m  No (ref.)  Yes | NI | NI | NI | NI | 1  0.95 (0.86–1.06) | 1  0.94 (0.83–1.07) | 1  1.60 (1.42–1.81)** | 1  1.26 (1.08–1.46)** |
| Facilities/amenities 800 m  0 (ref.)  1  2-3  > 4  P for trend | NI | NI | NI | NI | 1  1.13 (0.99–1.28)  1.08 (0.95–1.23)  1.06 (0.92–1.23)  0.534 | 1  1.14 (1.00–1.31)  1.12 (0.97–1.28)  1.11 (0.94–1.31)  0.386 | 1  1.32 (1.14–1.54)**  1.44 (1.23–1.68)**  1.24 (1.05–1.48)*  0.802 | 1  1.05 (0.90–1.24)  1.12 (0.94–1.33)  0.96 (0.79–1.17)  0.005 |
| Facilities/amenities 5 000 m  < 5 (ref.)  6-14  15-29  > 30  P for trend | NI | NI | NI | NI | 1  1.00 (0.86.–1.15)  1.04 (0.90–1.20)  1.05 (0.92–1.21)  0.333 | NE | 1  1.55 (1.32–1.82)**  1.76 (1.49–2.09)**  1.66 (1.42–1.93)**  0.491 | NE |
| Playgrounds/sports fields 800 m  < 1 (ref.)  2-5  6-10  > 11  P for trend | 1  0.90 (0.77–1.07)  0.95 (0.80–1.12)  0.84 (0.73–0.96)**  0.001 | NE | 1  0.91 (0.78-1.07)  0.99 (0.85–1.16)  0.84 (0.73–0.96)**  <0.001 | NE | 1  0.99 (0.83–1.17)  1.05 (0.88–1.25)  1.01 (0.88–1.17)  0.924 | NE | 1  2.76 (2.27–3.35)**  2.97 (2.44–3.62)**  2.98 (2.56–3.47)**  <0.001 | NE |
| Playgrounds/sports fields 5000 m  < 35 (ref.)  36-119  120-419  > 420  P for trend | NI | NI | NI | NI | 1  0.93 (0.81–1.07)  0.97 (0.84–1.11)  1.03 (0.89–1.19)  0.584 | NE | 1  1.90 (1.61–2.24)**  1.91 (1.62–2.26)**  1.94 (1.66–2.28)**  <0.001 | NE |
| School within 800 m  No  Yes | 1  0.92 (0.83–1.02) | 1  0.96 (0.86–1.07) | NI | NI | 1  1.07 (0.97–1.19) | 1  1.08 (0.97–1.22) | 1  1.15 (1.01–1.30)* | 1  0.93 (0.81–1.07) |
| School within 5 000 m  No  Yes | NI | NI | NI | NI | 1  1.08 (0.96–1.23) | 1  1.13 (0.98–1.30) | 1  1.68 (1.47–1.93)** | 1  1.16 (0.99–1.37) |
| Population density 800 m  < 200 (ref.)  201-799  800-1 649  > 1 650  P for trend | NI | NI | NI | NI | 1  0.96 (0.83–1.11)  1.04 (0.90–1.20)  0.96 (0.83–1.10)  0.302 | - | 1  2.32 (1.96–2.73)**  2.51 (2.13–2.96)**  1.95 (1.67–2.27)**  0.438 | - |
| Note: OR, odds ratio; PA, physical activity; NI, not included due to non-significance in bivariate models; NE, not estimated due to multicollinearity. *p<0.05. ** p<0.01.  ^a^ Adjusted for sex, participation in after-school care, mother’s age and level of education. ^b^ Additional adjustment for population density. | | | | | | | | |
